# Suspension of the New York College of Dentistry

**Published:** 1869-06

**Authors:** 


*Suspension of the New York College of Dentistry.*—We regret to learn that the "New York College of Dentistry" no longer exists, its charter having been annuled by the Supreme Court of the State in an action brought by the Attorney General. This action was instituted on a presentation of the condition of the College by a member of the faculty, who considered the acts of a majority of its Board of Trustees to be in direct violation of its charter.

The case came up before Judge Cardozo, and an injunction was granted and a receiver appointed to take charge of the property of the College on the 22nd of April last.

Previous to any steps being taken to annul its charter, no less than six of the most prominet members of its Board of Trustees resigned

As we are well acquainted with the untiring efforts of its former faculty to make it worthy the support of the profession, and of the pride they felt in its welfare, we deplore its fate and deeply sympathize with those who have expended so much time, labor and means in its behalf.

This is the second College of Dentistry which has been organized in the State of New York, and we had hoped that, unlike its predecessor, its career would be a long and prosperous one.

